# Supplementary material for: The scaling of social interactions across animal species
Source: Sci Rep. 2021 Jun 15;11:12584. doi: 10.1038/s41598-021-92025-1 (PMC8206375; doi:10.1038/s41598-021-92025-1)
Supplement: Supplementary file 1 — Supplementary Information. [file 41598_2021_92025_MOESM1_ESM.pdf]

Supplementary Information: The scaling of social interactions across animal species

Luis E C Rocha*
Department of Economics, Ghent University, Ghent, Belgium and
Department of Physics and Astronomy, Ghent University, Ghent, Belgium

Jan Ryckebusch
Department of Physics and Astronomy, Ghent University, Ghent, Belgium

Koen Schoors
Department of Economics, Ghent University, Ghent, Belgium

Matthew Smith
The Business School, Edinburgh Napier University, Edinburgh, UK
(Dated: May 21, 2021)

* luis.rocha@ugent.be

I. INTRODUCTION

This supplementary file contains a table with the number of network data sets and average group size per combination of social interaction and animal species. There are also information and references on the source of the data sets. The file contains the Python methods used in the network data analysis and the results of the analysis of the network structures for random versions of the original networks.

II. DATA COLLECTION

The data sets used in this study were collected using public network data repositories. Most animal social networks were extracted from the Animal Social Network Repository (https://github.com/bansallab/asnr/), most human social networks and some animal social networks came from The Colorado Index of Complex Networks (https://icon.colorado.edu), and human online friendship networks came from The Koblenz Network Collection (http://konect.uni-koblenz.de). A full list of references is available below organised by type of social interaction. For each reference, we also add an alias to help finding the network in the data file. Table I contains the number of networks for each species and type of social interactions together with the average number of nodes for each case.

TABLE I. Number of networks for each type of social interaction and animal class. Values in parentheses correspond to the mean number of nodes and respective standard deviation for each combination of species and social interaction. In a total of 611 networks, there are 179 cases of human and 432 cases of non-human social interactions, including 281 captive and 151 free-ranging animals.

Social interaction	Mammalian non-primates	Mammalian primates	Mammalian Humans	Actinopterygii	Aves	Insecta	Reptilia	Total
physical contact	0 (-)	4 (21±9)	0 (-)	0 (-)	2 (17 ±)	244 (104±35)	0 (-)	250
grooming	0 (-)	23 (15±6)	0 (-)	0 (-)	0 (-)	0 (-)	0 (-)	23
group membership	4 (33±4)	0 (-)	0 (-)	7 (10±0)	5 (41±14)	0 (-)	0 (-)	16
spatial proximity	63 (29±49)	58 (13±7)	88 (166±97)	9 (6±0)	0 (-)	12 (29±1)	1 (60±0)	231
offline friendship	0 (-)	0 (-)	67 (54±44)	0 (-)	0 (-)	0 (-)	0 (-)	67
online friendship	0 (-)	0 (-)	24 (4079019±13776026)	0 (-)	0 (-)	0 (-)	0 (-)	24
Total	67	85	179	16	7	256	1	611

A. Physical contact

Kutsukake2003 greeting

N Kutsukake, N Suetsugu and T Hasegawa. Pattern, distribution, and function of greeting behavior among black-and-white colobus. International Journal of Primatology 27(5):1271-1291 (2006)

Aves barnswallow association

II Levin et al. Stress response, gut microbial diversity and sexual signals correlate with social interactions. Biology Letters 12(6): 20160352 (2016)

Mammalia rhesusmacaque association

JJM Massen and EHM Sterck. Stability and durability of intra- and intersex social bonds of captive rhesus macaques (Macaca mulatta). International Journal of Primatology 34(4):770-791 (2013)

Insecta ants proximity

DP Mersch and A Crespi and L Keller. Tracking individuals shows spatial fidelity is a key regulator of ant social organization. Science 340(6136):1090-1093 (2013)

Mammalia spidermonkeys contact

R Rimbach et al. Brown spider monkeys (Ateles ¡br¿ hybridus): a model for differentiating the role of social networks and physical contact on parasite transmission dynamics. Philosophical Transactions Royal Society B 370(1669):20140110 (2015)

B. Grooming

baboons grooming t1 and t2
R Dunbar and P Dunbar. Social dynamics of gelada baboons. Contributions to Primatology 6:1 (1975)
Mammalia primates association
RH Griffin and CL Nunn. Community structure and the spread of infectious disease in primate social networks. Evolutionary Ecology 26(4):779-800 (2012)
Colobus monkeys
N Kutsukake, N Suetsugu and T Hasegawa. Pattern, distribution, and function of greeting behavior among black-and-white colobus. International Journal of Primatology 27(5):1271-1291 (2006)
Rhesus monkey grooming
DS Sade. Sociometrics of macaca mulatta I. linkages and cliques in grooming matrices. Folia Primatologica 18(3-4):196-223 (1972)

C. Group membership

Aves sparrow flockmembership
NN Arnberg et al. Social network structure in wintering golden-crowned sparrows is not correlated with kinship. Molecular ecology 24(19):5034-5044 (2015)
Actinopterygii fishstickleback
N Atton et al. Familiarity affects social network structure and discovery of prey patch locations in foraging stickleback shoals. Proceedings of the Royal Society of London B: Biological Sciences 281(1789):20140579 (2014)
Aves thornbill groupmembership
DR Farine, PJ Milburn. Social organisation of thornbill-dominated mixed-species flocks using social network analysis. Behavioral Ecology and Sociobiology 67(2):321-330 (2013)
Mammalia hyenas groupmembership
KE Holekamp et al. Society, demography and genetic structure in the spotted hyena. Molecular Ecology 21(3):613-632 (2012)
Aves sparrowlyon flockmembership
D Shizuka et al. Across-year social stability shapes network structure in wintering migrant sparrows. Ecology letters 17(8):998-1007 (2014)
Mammalia zebra groupmembership
SR Sundaresan, IR Fischhoff, J Dushoff and DI Rubenstein. Network metrics reveal differences in social organization between two fission-fusion species, Grevy's zebra and onager. Oecologia, 151(1):140-149 (2007)

D. Spatial proximity

Actinopterygii guppy
N Atton et al. Familiarity affects social network structure and discovery of prey patch locations in foraging stickleback shoals. Proceedings of the Royal Society of London B: Biological Sciences 281.1789:20140579 (2014)
Giraffe proximity
M Bashaw et al. The structure of social relationships among captive female giraffe (Giraffa camelopardalis). Journal Comparative Psychology 121(1):46-53 (2007)
Reptilia lizard
MC Bull, SS Godfrey and DM Gordon. Social networks and the spread of Salmonella in a sleepy lizard population. Molecular Ecology 21(17):4386-4392 (2012)
Haggle
A Chaintreau, P Hui, J Crowcroft, C Diot, R Gass and J Scott. Impact of human mobility on opportunistic forwarding algorithms. IEEE Transactions on Mobile Computing 6(6):606-620 (2007)
Reality Mining
N Eagle and A Pentland. Reality Mining: Sensing complex social systems. Personal Ubiquitous Computing 10(4):255-268 (2006)
Insecta beetle
V Formica et al. Consistency of animal social networks after disturbance. Behavioral Ecology arw128 (2016)
High school 2011 and 2012
J Fournet and A Barrat. Contact patterns among high school students. PLoS ONE 9(9):e107878 (2014)

Mammalia baboon association
M Franz, J Altmann and SC Alberts. Knockouts of high-ranking males have limited impact on baboon social networks. Current zoology 61(1):107-113 (2015)
Mammalia dolphin association
S Gazda et al. The importance of delineating networks by activity type in bottlenose dolphins (Tursiops truncatus) in Cedar Key, Florida. Royal Society open science 2(3):140263 (2015)
Primary School
V Gemmetto and A Barrat and C Cattuto. Mitigation of infectious disease at school: targeted class closure vs school closure. BMC Infectious Diseases 14:695 (2014)
tij InVS15 Workplace and SFHH Conference data
M Génois and A Barrat. Can co-location be used as a proxy for face-to-face contacts? EPJ Data Science 7:11 (2018)
tij InVS Workplace
M. Génois et al. Data on face-to-face contacts in an office building suggest a low-cost vaccination strategy based on community linkers. Network Science 3:326 (2015)
Mammalia kangeroos
TR Grant. Dominance and association among members of a captive and a free-ranging group of grey kangaroos (Macropus giganteus). Animal Behaviour 21(3):449-456 (1973)
INFECTIOUS cumulative daily networks and contact list
L Isella, J Stehlé, A Barrat, C Cattuto, J-F Pinton and Wouter Van den Broeck. What's in a crowd? Analysis of face-to-face behavioral networks. Journal of Theoretical Biology 271:166 (2011)
Kenyan households contact network
MC Kiti, M Tizzoni, TM Kinyanjui et al. Quantifying social contacts in a household setting of rural Kenya using wearable proximity sensors. EPJ Data Science 5:21 (2016)
High school contact network
R Mastrandrea, J Fournet and A Barrat. Contact patterns in a high school: a comparison between data collected using wearable sensors, contact diaries and friendship surveys. PLoS ONE 10(9):e0136497 (2015)
Mammalia raccoon
JJH Reynolds et al. Raccoon contact networks predict seasonal susceptibility to rabies outbreaks and limitations of vaccination. Journal of Animal Ecology 84(6):1720-1731 (2015)
Cows pen 4 and pen 5
LEC Rocha, O Terenius, I Veissier, B Meunier, PP Nielsen. Persistence of sociality in group dynamics of dairy cattle. Applied Animal Behaviour Science 223:104921 (2020)
Howler spatial proximity
L Sailer and S Gaulin. Proximity, sociality, and observation: the definition of social groups. American Anthropologist 86(1):91-98 (1984)
Sapiezynski
P Sapiezynski, A Stopczynski, DD Lassen et al. Interaction data from the Copenhagen Networks Study. Scientific Data 6:315 (2019)
sp data school day 1 g and day 2 g
J Stehlé, N Voirin, A Barrat, C Cattuto, L Isella, J-F Pinton, M Quaggiotto, W Van den Broeck, C Régis, B Lina and P Vanhems. PLOS ONE 6(8):e23176 (2011)
detailed list of contacts Hospital
P Vanhems et al. Estimating Potential Infection Transmission Routes in Hospital Wards Using Wearable Proximity Sensors. PLoS ONE 8(9):e73970 (2013)
Dog spatial proximity 1 and 2
JK Wilson-Aggarwal, L Ozella, M Tizzoni, C Cattuto, GJF Swan, T Moundai et al. High-resolution contact networks of free-ranging domestic dogs Canis familiaris and implications for transmission of infection. PLoS Neglected Tropical Diseases 13(7):e0007565 (2019)

E. Offline Friendship

van de Bunt's freshmen
G van de Bunt et al. Friendship Networks Through Time: An Actor-Oriented Dynamic Statistical Network Model. Computational & Mathematical Organization Theory 5(2), 167-192 (1999)
Zeggelink's freshmen
G Van de Bunt, M Van Duijn and T Snijders Friendship networks through time: An actor-oriented dynamic statistical network model. Computational & Mathematical Organization Theory 5.2. 167-192. (1999)

Haggle human proximity network
A Chaintreau et al. "Impact of human mobility on opportunistic forwarding algorithms." IEEE Trans. on Mobile Computing 6(6), 606-620 (2007)
Illinois high school students
JS Coleman. Introduction to Mathematical Sociology. London Free Press Glencoe (1964)
Reality mining proximity network
N Eagle and A Pentland. Reality mining: sensing complex social systems. Personal and Ubiquitous Computing 10(4), 255-268 (2006)
Student cooperation
M Fire et al. Predicting student exam's scores by analyzing social network data. International Conference on Active Media Technology, 584-595 (AMT 2012)
ANU Residence Hall network
L Freeman, C Webster and D Kirke. Exploring social structure using dynamic three-dimensional color images. Social Networks 20(2), 109-118 (1998)
London street gang
T Grund, and J Densley. Ethnic homophily and triad closure: Mapping internal gang structure using exponential random graph models. Journal of Contemporary Criminal Justice 31(3), 354-370 (2015)
Dutch 3rd grade friendships
B Houtzager and C Baerveldt. Just like normal: A social network study of the relation between petty crime and the intimacy of adolescent friendships. Social Behavior and Personality: An International Journal 27(2), 177-192 (1999)
Kapferer tailor shop
B Kapferer. Strategy and transaction in an African factory. Manchester University Press (1972)
Kenyan households contacts
M Kiti et al. Quantifying social contacts in a household setting of rural Kenya using wearable proximity sensors. EPJ Data Science 5, 21 (2016)
Knecht Dutch school
A Knecht. Friendship selection and friends' influence. Dynamics of Networks and Actor Attributes in Early Adolescence. PhD Dissertation, Utrecht University (2008)
Lazega law firm network
E Lazega. The Collegial Phenomenon: The Social Mechanisms of Cooperation Among Peers in a Corporate Law Partnership. Oxford University Press (2001)
Teenage Friends and Lifestyle Study
L Michell and A Amos. Girls, pecking order and smoking. Social Science & Medicine 44(12), 1861-1869 (1997)
Gagnon and MacRae prison
D MacRae. Direct factor analysis of sociometric data. Sociometry 23(4), 360-371 (1960)
Aarhus Computer Science department relationships
M Magnani, B Micenkova, and L Ross. Combinatorial Analysis of Multiple Networks. arXiv:1303.4986 (2013)
Parker and Asher's friendships
JG Parker and SR Asher. Friendship and friendship quality in middle childhood: Links with peer group acceptance and feelings of loneliness and social dissatisfaction. Developmental Psychology 29(4), 611 (1993)
Robins bankers
P Pattison at al. Statistical evaluation of algebraic constraints for social networks. Journal of Mathematical Psychology 44(4), 536-568 (2000)
Sampson's monastery
SF Sampson. A Novitiate in a Period of Change. An Experimental and Case Study of Social Relationships. PhD thesis Cornell University (1968)
Copenhagen Networks Study
P Sapiezynski et al. Interaction data from the Copenhagen Networks Study. Scientific Data 6, 315 (2019)
Dutch school friendships
T Snijders, G van de Bunt and C Steglich. Introduction to stochastic actor-based models for network dynamics. Social Networks 32(1), 44-60 (2010)
Vickers 7th Graders
M Vickers and S Chan. Representing Classroom Social Structure. Melbourne: Victoria Institute of Secondary Education, (1981)
Zachary Karate Club
WW Zachary. An information flow model for conflict and fission in small groups. Journal of Anthropological Research 33, 452-473 (1977)

Hartford drugnet
M Weeks, et al. Social networks of drug users in high-risk sites: Finding the connections. AIDS and Behavior 6(2), 193-206 (2002)

F. Online Friendship

Catster
Catster friendships network dataset – KONECT, April 2017
Gowalla and Brightkite
E Cho, SA Myers, and J Leskovec. Friendship and mobility: User movement in location-based social networks. In Proc. Int. Conf. on Knowledge Discovery and Data Mining, pages 1082–1090, 2011
Dogster
Dogster friendships network dataset – KONECT, April 2017
Friendster
Friendster network dataset – KONECT, April 2017
Hamsterster full
Hamsterster full network dataset – KONECT, April 2017
Hamsterster
Hamsterster friendships network dataset – KONECT, April 2017
Digg
T Hogg and K Lerman. Social dynamics of Digg. EPJ Data Science, 1(5), 2012
Livejournal
J Leskovec, KJ Lang, A Dasgupta, and MW Mahoney. Statistical properties of community structure in large social and information networks. In Proc. Int. World Wide Web Conf., pages 695–704, 2008
Google+ and facebook nips
J McAuley and J Leskovec. Learning to discover social circles in ego networks. In Advances in Neural Information Processing Systems, pages 548–556. 2012
Youtube
A Mislove. Online Social Networks: Measurement, Analysis, and Applications to Distributed Information Systems. PhD thesis, Rice University, 2009
Flickr
A Mislove, HS Koppula, KP Gummadi, P Druschel, and B Bhattacharjee. Growth of the Flickr social network. In Proc. Workshop on Online Social Networks, pages 25–30, 2008
Orkut, Liverjournal links, Flickr links, and youtube links
A Mislove, M Marcon, KP Gummadi, P Druschel, and B Bhattacharjee. Measurement and analysis of online social networks. In Proc. Internet Measurement Conf., 2007
Filmtipset
A Said, EW De Luca, and S Albayrak. How social relationships affect user similarities. In Proc. IUI Workshop on Social Recommender Systems, 2010
Pokec
L Takac and M Zabovsky. Data analysis in public social networks. In Int. Scientific Conf. and Int. Workshop Present Day Trends of Innovations, 2012
Facebook friendships
B Viswanath, A Mislove, M Cha, and KP Gummadi. On the evolution of user interaction in Facebook. In Proc. Workshop on Online Social Networks, pages 37–42, 2009
Youtube friendship
J Yang and J Leskovec. Defining and evaluating network communities based on ground-truth. In Proc. ACM SIGKDD Workshop on Mining Data Semantics, page 3. ACM, 2012
Hyves, Flixster, and Livemocha
R Zafarani and H Liu. Social computing data repository at ASU, 2009

III. DATA PROCESSING

All data sets were curated, including the removal of self-loops, edge directions, edge weights, edge timings, and analysed using JupyterLab, Python 3.6.4 programming language, NetworkX 2.4 library for network analysis, and seaborn 0.10.1 library for statistical data visualisation. Key functions are shown in Table II

TABLE II. NetworkX functions used to calculate network characteristics.

Function	Code
Make the network undirected	Graph.to_undirected()
Remove self-loops	Graph.remove_edges_from(nx.selfloop_edges(Graph))
Count number of nodes	Graph.number_of_nodes()
Count number of edges	Graph.number_of_edges()
Calculate average clustering coefficient	nx.average_clustering(Graph)
Calculate average shortest-path length of the largest connected component	Gcc = sorted(nx.connected_components(Graph), key=len, reverse=True) nx.average_shortest_path_length(Graph.subgraph(Gcc[0]))

IV. RANDOM NETWORK STRUCTURES

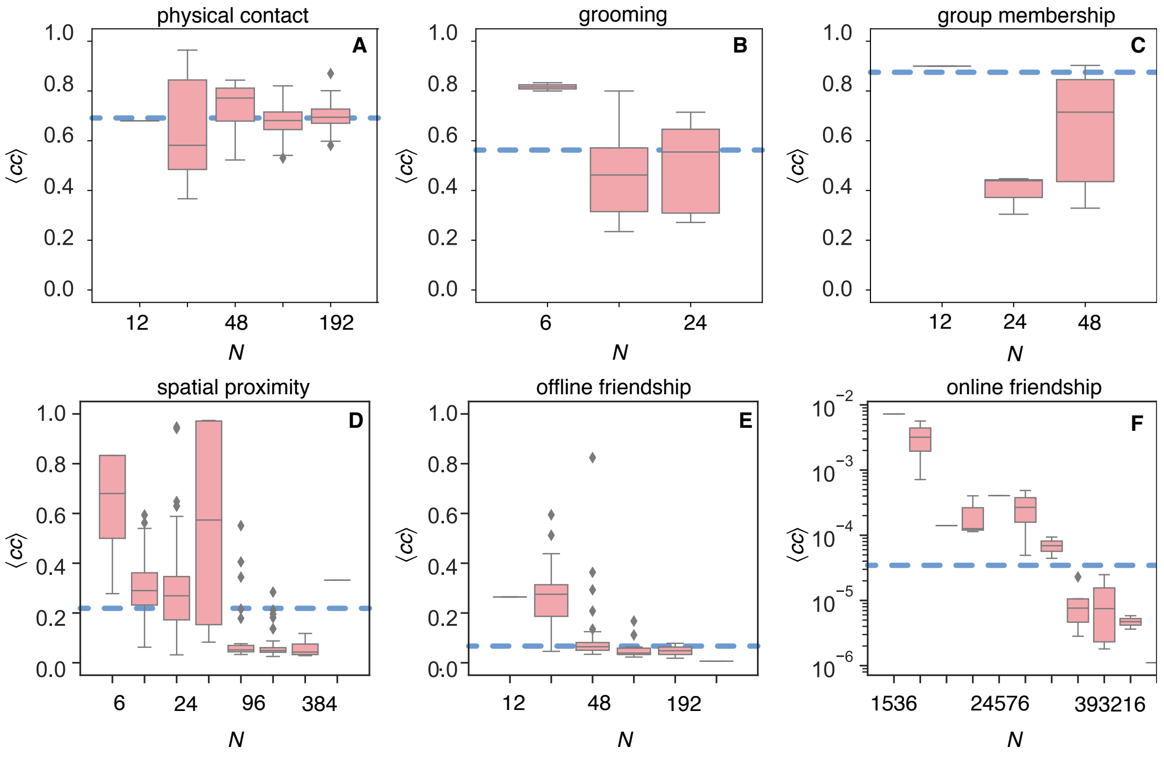

FIG. 1. Network clustering structures for the random version of the networks. The average clustering coefficient $\langle cc \rangle$ between close contacts vs. network size for (A) physical contact (Median $M_{\text{rand}} = 0.88$); (B) grooming ($M_{\text{rand}} = 0.56$); (C) group membership ($M_{\text{rand}} = 0.69$); (D) spatial proximity ($M_{\text{rand}} = 0.22$); (E) offline friendship ($M_{\text{rand}} = 0.07$); (F) online friendship ($M_{\text{rand}} = 3.5 \cdot 10^{-5}$). Dashed horizontal lines are the median values of the empirical networks. Log-binned (x-axes) Tukey box plots with diamonds representing outliers.

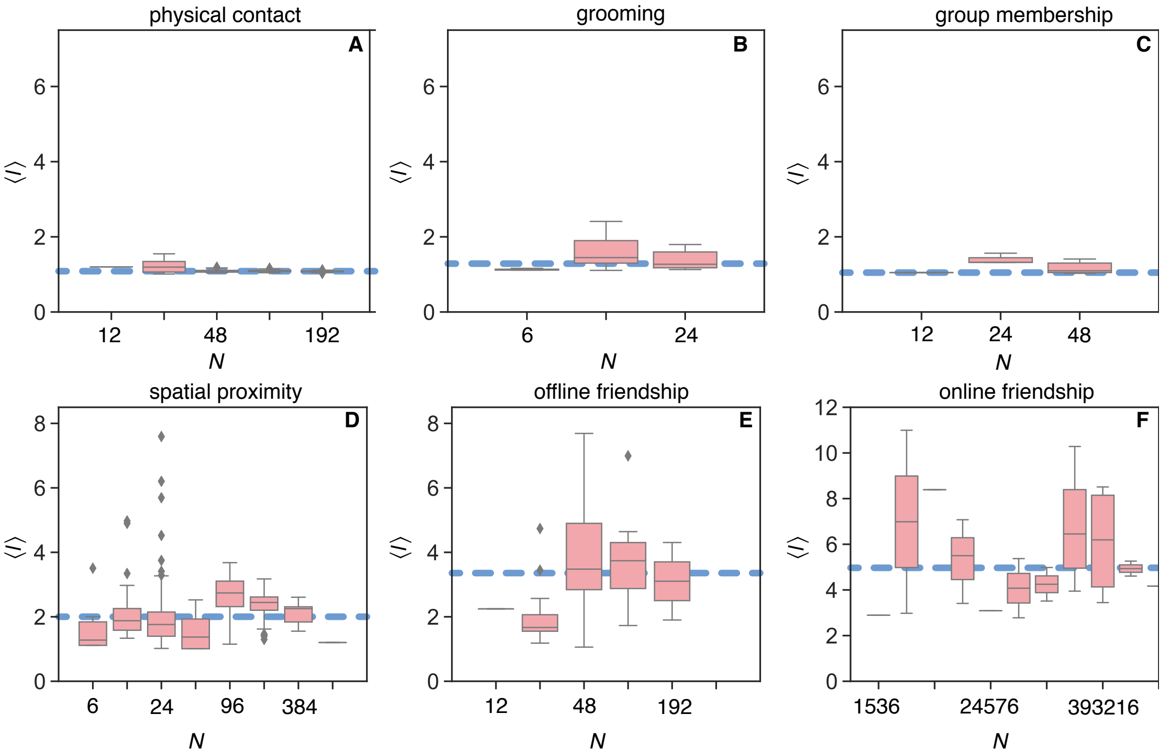

FIG. 2. Network path structures for the random version of the networks. The average shortest path-length $\langle l \rangle$ vs. network size in the networks of (A) physical contact (Median $M_{\mathrm{rand}} = 1.05$); (B) grooming ($M_{\mathrm{rand}} = 1.29$); (C) group membership ($M_{\mathrm{rand}} = 1.09$); (D) spatial proximity ($M_{\mathrm{rand}} = 2.00$); (E) offline friendship ($M_{\mathrm{rand}} = 3.36$); (F) online friendship ($M_{\mathrm{rand}} = 4.97$). Dashed horizontal lines are the median values of the empirical networks. Log-binned (x-axes) Tukey box plots with diamonds representing outliers.
